# Associations Between Cancer-Related Self-Efficacy, Coping Styles, and Perceived Stress in Patients with Lung Cancer: A Cross-Sectional Mediation Analysis

**DOI:** 10.3390/jcm15176674

**Published:** 2026-08-28

**Authors:** Agata Poręba-Chabros, Magdalena Kolańska-Stronka, Marzanna Farnicka

**Affiliations:** 1 Institute of Psychology, John Paul II Catholic University of Lublin, Al. Racławickie 14, 20-950 Lublin, Poland; aporebachabros@gmail.com; 2 Institute of Psychology, SWPS University, Chodakowska 19/31, 03-815 Warsaw, Poland; 3 Institute of Social Prevention and Resocialization, University of Warsaw, ul. Krakowskie Przedmieście Street 26/28, 00-927 Warsaw, Poland; m.farnicka@uw.edu.pl

**Keywords:** lung cancer, cancer-related self-efficacy, perceived stress, coping styles

## Abstract

Lung cancer carries a substantial psychosocial burden, frequently involving elevated psychological distress, disease-related stigma, and difficulties in adaptation to illness. Coping styles and self-efficacy are both recognized as relevant personal resources in chronic illness, but their combined statistical association with perceived stress, and specifically whether self-efficacy accounts for part of the association between coping and stress, has not been examined in lung cancer patients. **Objective**: This study examined the statistical associations between coping styles (task-, emotion-, and avoidance-oriented), cancer-related self-efficacy, and perceived stress, testing cancer-related self-efficacy as a mediator of each coping style’s association with perceived stress, and additionally compared these variables by gender. **Methods**: A cross-sectional study was conducted among ninety-seven patients diagnosed with lung cancer. Standardized measures included the Coping Inventory for Stressful Situations, a cancer-specific adaptation of the Generalized Self-Efficacy Scale, and the Perceived Stress Scale. A series of three simple mediation analyses was performed to evaluate the proposed statistical model. **Results**: Task-oriented coping was positively, and emotion-oriented coping negatively, associated with cancer-related self-efficacy; avoidance-oriented coping showed no significant association with self-efficacy. Task- and emotion-oriented coping were significantly associated with perceived stress in the expected directions, while avoidance-oriented coping was not. Cancer-related self-efficacy significantly, and partially, mediated the association between task-oriented coping and perceived stress; no significant indirect effect was found for emotion- or avoidance-oriented coping. Women reported significantly higher perceived stress and greater use of emotion-oriented coping than men, with no significant gender differences in self-efficacy or the other coping styles. **Conclusions**: In this cross-sectional sample, cancer-related self-efficacy was statistically associated with lower perceived stress specifically through its association with task-oriented coping, whereas the association between emotion-oriented coping and stress appeared largely independent of self-efficacy. These findings suggest that coping styles are associated with perceived stress through at least partly distinct mechanisms and support attention to both active coping and self-efficacy beliefs in psychosocial care for lung cancer patients, while findings regarding gender differences warrant further, formally tested investigation.

## 1. Introduction

Lung cancer represents a unique oncological challenge not only due to its high mortality rates and frequently unfavorable prognosis but also because of the specific psychosocial burden associated with the diagnosis. Compared with patients affected by other types of cancer, individuals with lung cancer often experience distinct psychological difficulties, including elevated distress, reduced quality of life, and impaired adaptation to illness [1]. Indeed, empirical evidence confirms that the prevalence and severity of psychological distress vary significantly across oncological diagnoses, with lung cancer patients consistently demonstrating some of the highest levels of emotional burden [2].

The psychological burden of lung cancer is not merely a subjective experience but is paralleled by measurable biological stress responses. Newly diagnosed patients frequently report acute traumatic stress [3], and elevated stress-related neuroendocrine markers have been observed even prior to diagnosis in individuals later found to have lung cancer, compared with those with benign pulmonary pathology [4]. Preclinical evidence further indicates that chronic psychological stress may accelerate tumor growth and metastatic spread through neuroendocrine and immune-mediated pathways, including β-adrenergic signaling and stress-induced remodeling of the pulmonary microenvironment [5,6]. These findings underscore that perceived stress in lung cancer patients carries potential biological as well as psychosocial significance, reinforcing the clinical relevance of identifying modifiable psychological resources, such as coping and self-efficacy, that may help regulate stress in this population.

A particularly important factor influencing patients’ psychological adjustment is disease-related stigma. Because lung cancer is strongly associated with tobacco smoking, patients frequently encounter social judgments and may experience guilt or self-blame regarding their illness. Such experiences may reduce perceived social support and intensify emotional distress [1]. Previous reports indicate that a substantial proportion of lung cancer patients experience clinically significant psychological distress, including symptoms of anxiety and depression [1]. Additionally, concerns regarding disease progression, painful death, reduced physical functioning, and frequent comorbidities further contribute to difficulties in adaptation. In particular, the experience of cancer-related pain is not merely a somatic symptom; it induces functional and structural changes in the central nervous system that actively generate negative affective states, thereby compounding the psychological burden [7].

The psychological response to lung cancer is also shaped by individual characteristics and coping processes. Recent findings suggest relevant gender differences in emotional adjustment, with women generally reporting higher levels of anxiety and depressive symptoms, whereas men more frequently demonstrate apathy or externalizing responses [8,9]. Furthermore, gender acts as a critical determinant in how patients experience symptoms, as women and men systematically differ not only in their pain perception but also in the specific coping strategies they deploy to manage oncological stress [10]. Among lung cancer outpatients, the utilization of specific, often maladaptive or repressive coping styles has been directly linked to higher levels of depression and exacerbated pain severity [11]. Moreover, lung cancer is associated with considerable social and occupational consequences, including a higher likelihood of employment loss and transition to disability compared with several other cancer populations [12,13]. Therefore, identifying psychological resources that facilitate adaptation to this disease remains an important objective in psycho-oncological research.

One personal resource consistently associated with adaptation to chronic illness is self-efficacy. According to Bandura’s [14] social cognitive theory, self-efficacy reflects individuals’ beliefs regarding their ability to organize and execute the courses of action required to manage prospective situations. Because self-efficacy beliefs are domain-specific, research on chronic illness has increasingly relied on illness-tailored adaptations of general self-efficacy measures to capture patients’ confidence in managing disease-specific demands, rather than their generalized sense of competence [15,16]. In the present study, self-efficacy is therefore conceptualized and measured as cancer-related self-efficacy—patients’ confidence in their ability to cope with the specific demands imposed by lung cancer and its treatment—rather than as generalized self-efficacy in Bandura’s original sense. Stronger cancer-related self-efficacy beliefs have been associated with better psychological functioning, higher quality of life, and lower levels of anxiety and depression among cancer patients [15,17,18]. Self-efficacy has further been shown to partially mediate the association between broader adjustment resources and health-related quality of life [19], and, among children undergoing cancer treatment, to exert the largest total effect on psychological stress among the personal resources examined [20]. From the perspective of the Conservation of Resources theory [21], self-efficacy may function as a key psychological resource: individuals who believe in their capacity to manage illness-related demands are better positioned to acquire and protect additional resources when confronted with the stress of cancer, whereas those with lower self-efficacy are more vulnerable to resource loss spirals that intensify distress.

Self-efficacy alone, however, may not fully account for individual differences in psychological adaptation. According to the transactional model of stress and coping proposed by Lazarus and Folkman [22], the way a stressor affects well-being depends on how it is cognitively appraised and on the coping efforts mobilized in response. Coping styles—relatively stable dispositional tendencies in how individuals respond to stressful situations, as opposed to situation-specific coping strategies—may therefore represent a proximal resource that shapes such appraisals and, in turn, personal resources such as self-efficacy [23,24]. Three coping styles are typically distinguished in this literature: task-oriented coping, involving active problem management and direct engagement with the stressor; emotion-oriented coping, characterized by a focus on one’s own emotional reactions and negative affect; and avoidance-oriented coping, involving cognitive or behavioral disengagement from the stressor, including distraction and seeking social diversion. Task-oriented coping is generally considered the most adaptive of the three, whereas emotion-oriented coping has been consistently associated with higher distress and poorer psychological adjustment among cancer patients [25,26]. The role of avoidance-oriented coping is less consistent: it has been linked to poorer adjustment and higher depression and pain in several studies [11,25,26], yet other evidence suggests it may offer short-term buffering against acute stress depending on the controllability of the situation and its distraction-related components, making its association with outcomes more context-dependent than that of task- or emotion-oriented coping. This framework continues to receive contemporary empirical support, confirming that cognitive appraisal and coping mechanisms play an important role in psychological and psychosomatic adaptation to severe health stressors [24]. The association between coping and self-efficacy is well established empirically, but the literature is not unanimous regarding its direction. Several studies have modeled self-efficacy as an antecedent that shapes subsequent coping: in a structural equation model of Chinese cancer survivors, self-efficacy exerted both direct and indirect effects on self-management behaviors through coping style [27], and in a prospective study of women with breast cancer, baseline coping self-efficacy predicted patients’ coping reactions three months later, although hopelessness/helplessness in turn predicted subsequent declines in self-efficacy, suggesting the relationship may be reciprocal over time [28]. Other cross-sectional evidence, however, is consistent with the reverse pathway: self-efficacy has been found to be moderately, positively correlated with task-oriented coping and inversely correlated with emotion-oriented coping [29], a pattern consistent with active, task-oriented coping efforts functioning as a source of self-efficacy beliefs rather than merely their consequence. Directly relevant to the present population, self-care self-efficacy and positive coping have been shown to operate as sequential mediators between social support and psychological resilience among patients undergoing treatment for lung cancer specifically [30]—the only external study identified that examined self-efficacy and coping jointly in this diagnostic group. Because the available cross-sectional and longitudinal evidence does not converge on a single causal ordering, the present study adopts a theoretically motivated position: following Bandura’s [14] account of enactive mastery experience as the strongest source of self-efficacy beliefs, coping styles are modeled here as a proximal resource associated with cancer-related self-efficacy, rather than as its downstream consequence. This choice is revisited as a limitation in the Discussion, given that the cross-sectional design cannot rule out the reverse or a reciprocal pathway.

The plausibility of coping as a proximal determinant of psychological adjustment in lung cancer is further supported by prior work from our own group using the same clinical sample examined in the present study. In this cohort of 97 patients with lung cancer, task-oriented coping was negatively, and emotion-oriented coping was positively, associated with perceived stress, and perceived stress fully mediated the association between emotion-oriented coping and pain [31]. A separate analysis of the same sample showed that coping styles—particularly emotion-oriented coping—mediated the association between patients’ cognitive appraisal of their disease and their perceived stress level [32]. Notably, in the first of these studies the moderating role of gender on the coping–stress pathway was only marginal, with a 95% confidence interval for the index of moderated mediation crossing zero [31]. A third analysis of this sample identified social support as a further moderator of the appraisal–stress relationship, with informational support unexpectedly associated with higher, rather than lower, perceived stress [33]—indicating that social support is a further psychosocial resource relevant to stress in this population that was not the focus of, and was not controlled for in, the present analysis. Building on this established role of coping styles in shaping perceived stress in this specific clinical population, the present study extends this line of research by examining cancer-related self-efficacy as a previously untested mechanism through which coping styles may be associated with perceived stress.

Although previous studies have demonstrated associations between self-efficacy, coping strategies, and psychological outcomes among oncology patients [16,27,30], the mechanisms underlying these relationships remain insufficiently understood. Existing research has predominantly focused on direct associations between self-efficacy, symptom distress, and overall well-being, often without integrating coping styles as proximal determinants of self-efficacy [18,34]. Consequently, limited evidence exists regarding whether cancer-related self-efficacy represents a pathway through which task-, emotion-, and avoidance-oriented coping are associated with perceived stress among patients with lung cancer.

### The Present Study

Given the identified gap in the literature, the present study aimed to examine a theoretical model of psychological adaptation among patients with lung cancer. Specifically, the study investigated the relationships between coping styles (task-, emotion-, and avoidance-oriented), cancer-related self-efficacy, and perceived stress, with particular attention to the potential mediating role of cancer-related self-efficacy in the association between coping styles and perceived stress. Given previously reported gender differences in psychological adjustment to cancer [8,9,10], and given the marginal, non-significant moderating role of gender identified in our prior work on this same sample [31], gender was not included as a covariate or moderator within the mediation model; instead, potential gender differences in coping styles, cancer-related self-efficacy, and perceived stress were examined separately through between-group comparisons, reported alongside the main mediation analysis. Within Lazarus and Folkman’s [22] transactional framework and Bandura [14] social cognitive theory, task-oriented coping—reflecting active engagement with cancer-related demands—is expected to function as a mastery experience that strengthens cancer-related self-efficacy, whereas emotion-oriented and avoidance-oriented coping are expected to be associated with lower self-efficacy. Consistent with Conservation of Resources theory [21], coping efforts represent a basic resource that can generate further resources—including self-efficacy beliefs—in a resource caravan; individuals who mobilize task-oriented coping are thus expected to accumulate the resource of self-efficacy, which in turn is expected to be associated with lower perceived stress. This proposed pathway is further motivated by evidence that perceived stress in lung cancer patients is not only a subjective experience but is paralleled by measurable psychobiological stress responses [3,4], and, in preclinical models, by biological pathways through which chronic stress may influence tumor behavior [5,6]. These findings reinforce the clinical relevance of identifying the resources—such as active, task-oriented coping and the self-efficacy beliefs it may support—that are associated with lower perceived stress in this population.

On this basis, the following hypotheses were formulated:

**H1.**  *Coping styles were expected to be associated with stress level. Specifically:*

**H1a.**  *Task-oriented coping will be negatively associated with perceived stress;*

**H1b.**  *Emotion-oriented coping will be positively associated with perceived stress;*

**H1c.**  *Avoidance-oriented coping will be positively associated with perceived stress.*

**H2.**  *Coping styles will be associated with cancer-related self-efficacy among individuals with cancer. Specifically:*

**H2a.**  *Task-oriented coping will be positively associated with cancer-related self-efficacy;*

**H2b.**  *Emotion-oriented coping will be negatively associated with cancer-related self-efficacy;*

**H2c.**  *Avoidance-oriented coping will be negatively associated with cancer-related self-efficacy.*

**H3.**  *Cancer-related self-efficacy was expected to be negatively associated with perceived stress.*

**H4.**  *Cancer-related self-efficacy was expected to statistically mediate the associations between coping styles and perceived stress.*

Given the cross-sectional design of the study, these hypotheses describe expected patterns of association rather than causal or temporal sequences. As noted above, cross-sectional and longitudinal evidence in the broader oncology literature is not unanimous regarding the direction of the coping–self-efficacy association [27,28,29]; the statistical mediation tested in H4 refers to an indirect association identified in cross-sectional data and does not establish that coping styles precede self-efficacy, or that self-efficacy precedes perceived stress, in time. Alternative orderings, including a reciprocal relationship between coping and self-efficacy, remain plausible and are addressed in the Section 4.

## 2. Materials and Methods

### 2.1. Participants and Procedure

Prior to data collection, the study protocol was approved by the University Ethical Board and conducted in accordance with the ethical principles outlined in the Declaration of Helsinki (1964) and its subsequent amendments (approval no. 02/06/16). The study was carried out at the Independent Public Clinical Hospital No. 4 in Lublin, Poland, following approval from the hospital administration, the head of the oncology department, and the hospital ethics committee. Data were collected between October 2016 and September 2017.

Patients were eligible for inclusion if they had a confirmed diagnosis of lung cancer; no additional inclusion or exclusion criteria (e.g., regarding age, disease stage, treatment phase, or psychiatric comorbidity) were applied. Patients were recruited from among those receiving care at the above department during the recruitment period. Of 100 patients invited to participate, 3 declined (non-response rate = 3%), yielding a final analytic sample of 97 patients.

The final sample consisted of 97 patients diagnosed with lung cancer, including 50 men (51.5%) and 47 women (48.5%). Participants ranged in age from 35 to 84 years (*M* = 64.84, *SD* = 7.82). Full sociodemographic and clinical characteristics of the sample are presented in Table 1. Data on disease stage, metastatic status, and treatment line were not collected; this is acknowledged as a limitation in Section 5. Smoking status was assessed as current smoking only and did not distinguish former smokers from never-smokers within the non-smoking group.

Data were collected through individual, paper-and-pencil, face-to-face sessions conducted directly with patients by a researcher trained in communicating with oncology patients. Questionnaire items and response options were read aloud to each participant by the researcher rather than completed independently; this procedure was adopted given the clinical characteristics of the sample, including advanced age and disease-related physical burden, and was intended to reduce the risk of comprehension or completion difficulties. Participation was voluntary, and all participants provided informed consent prior to completing the study materials. Participants were informed about the purpose of the research, the anonymous nature of data collection, and their right to withdraw at any time without consequences. The consent form included contact information for the principal investigator and stated that completion of the questionnaire constituted informed consent to participate in the study. Each individual session lasted approximately 30 to 60 min and was conducted in a private setting within the hospital to ensure confidentiality and participant comfort.

### 2.2. Missing Data

Missing data across study variables were minimal, accounting for approximately 2% of all recorded data points, and did not show evidence of a systematic pattern related to the study variables. Given this low proportion, pairwise deletion was used in all correlational and mediation analyses, and no data imputation was performed.

### 2.3. Measures

Three standardized instruments were used in the study.

***Cancer-related self-efficacy.*** Cancer-related self-efficacy was assessed using an adapted version of the Generalized Self-Efficacy Scale (GSES), developed by Schwarzer and Jerusalem [35] and adapted into Polish [36]. The original GSES measures individuals’ general beliefs about their ability to cope with difficult situations and demands. In the present study, all items were modified to refer specifically to coping with cancer-related situations (e.g., “I can usually handle difficult problems related to my illness if I try hard enough”). Thus, the adapted version assessed participants’ perceived ability to effectively manage challenges associated with cancer and its treatment. The scale consists of 10 items rated on a 4-point Likert scale ranging from 1 (not true at all) to 4 (exactly true). The total score is calculated as the sum of all items, with higher scores indicating higher perceived self-efficacy. To evaluate whether the modified, cancer-related items of the Generalized Self-Efficacy Scale [35,36] represented a unidimensional construct, a confirmatory factor analysis (CFA) was conducted on the 10 items using maximum likelihood estimation. The hypothesized one-factor model, with four pairs of correlated item residuals added based on modification indices, showed acceptable fit on most indices, χ^2^(31) = 88.2, *p* < 0.001, CFI = 0.931, TLI = 0.900, SRMR = 0.047, but an elevated RMSEA, 0.138, 90% CI [0.105, 0.172]. The elevated RMSEA is consistent with its known tendency toward inflation in models with a small number of degrees of freedom and modest sample size. All standardized factor loadings were statistically significant and ranged from 0.49 to 0.92 (all *p*s < 0.001; Appendix A), supporting the item set as reflecting a single underlying construct. The internal consistency of the scale in the present study was high, with Cronbach’s α = 0.93.

***Styles of coping with stress***. The Coping Inventory for Stressful Situations (CISS) developed by Endler and Parker [37] was used to measure coping styles. It consists of 48 statements that represent different behaviours activated in stressful situations. The questionnaire consists of three subscales corresponding to three coping styles: (TOS) task-oriented style; (EOS) emotion-oriented style; and (AOS) avoidance-oriented style. Each of these subscales consists of 16 items, and the respondents can score from 16 to 80 points in each. In the present study, the scale demonstrated high internal consistency, with Cronbach’s reliability coefficients (alpha) ranging from 0.84 for the emotion-oriented style subscale to 0.90 for the task-oriented style subscale.

***Stress level***. The Perceived Stress Scale (PSS-10) developed by Cohen et al. was used to measure the stress level variable [38,39]. The scale was designed to assess an individual’s response to a stressful situation in which they find themselves. The total scale score is the sum of all items and can take values from 0 to 40, with higher scores indicating higher perceived stress. In the case of the stress scale, the results in this study were obtained by calculating the total value obtained for all questions. In the present study, Cronbach’s internal reliability coefficient α is high and stands at 0.91.

### 2.4. Statistical Analysis

Statistical analyses were performed using IBM SPSS Statistics version 28.0. Descriptive statistics (means, standard deviations, medians, ranges, skewness, and kurtosis) were calculated for all study variables. Distributional properties were examined using skewness and kurtosis coefficients, which indicated no substantial deviations from normality and supported the use of parametric statistical procedures.

Bivariate relationships among cancer-related self-efficacy, coping styles, and perceived stress were examined using Pearson product-moment correlation coefficients. The 95% confidence intervals for each correlation coefficient were estimated using a bootstrap procedure with 1000 resamples. Gender differences in the study variables were assessed using independent-samples *t*-tests. Effect sizes were calculated using Cohen’s *d* and interpreted according to Cohen’s [40] guidelines as small (0.20), medium (0.50), and large (0.80).

To examine the mediating role of cancer-related self-efficacy in the relationship between coping styles and perceived stress, a series of simple mediation analyses was conducted using Hayes’ PROCESS macro for SPSS (Model 4; [41]). Coping styles were entered as the independent variable, perceived stress as the dependent variable, and cancer-related self-efficacy as the mediator. The significance of indirect effects was evaluated using a bias-corrected bootstrap procedure with 5000 resamples. Indirect effects were considered statistically significant when the 95% bootstrap confidence interval did not include zero. All statistical tests were two-tailed, and the significance level was set at α = 0.05.

Artificial intelligence (AI) tools were used exclusively to assist with language translation and editorial refinement of the manuscript. AI support was limited to improving grammar, clarity, and linguistic accuracy. The authors take full responsibility for the content and integrity of the manuscript.

## 3. Results

### 3.1. Descriptive Analyses

Descriptive statistics for all study variables are presented in Table 2.

The mean level of perceived stress was *M* = 17.33 (*SD* = 6.60), with scores ranging from 2 to 37. The mean score for cancer-related self-efficacy was *M* = 29.24 (*SD* = 6.03).

Regarding coping styles, participants reported the highest use of task-oriented coping (*M* = 57.30, *SD* = 8.18), followed by avoidance-oriented coping (*M* = 44.89, *SD* = 6.00) and emotion-oriented coping (*M* = 38.66, *SD* = 8.91). Median values were comparable to the corresponding means across all variables, suggesting relatively balanced distributions.

Inspection of distributional properties indicated that most variables approximated normality. Cancer-related self-efficacy exhibited moderate negative skewness (*Sk* = −0.76), suggesting a tendency toward higher self-efficacy scores. The remaining variables showed only slight departures from symmetry, with skewness values ranging from 0.03 to 0.38. Kurtosis values ranged from −0.20 to 1.87. The highest kurtosis was observed for cancer-related self-efficacy (*Kurt* = 1.87), indicating a somewhat more peaked distribution, whereas the coping styles and perceived stress demonstrated kurtosis values close to zero, suggesting distributions that did not substantially deviate from normality. Overall, the observed skewness and kurtosis coefficients supported the use of parametric statistical procedures.

### 3.2. Gender Differences in Study Variables

Independent-samples *t*-tests were conducted to examine gender differences in the study variables. Levene’s tests indicated homogeneity of variances for all variables (*p* > 0.05).

The analyses revealed significant gender differences in perceived stress and emotion-oriented coping. Women reported higher levels of perceived stress and greater use of emotion-oriented coping strategies than men. Both differences were associated with moderate effect sizes (*d* = 0.70 and *d* = 0.72, respectively).

No significant gender differences were observed for cancer- related self-efficacy, task-oriented coping, or avoidance-oriented coping (*ps* > 0.05). Although men scored slightly higher on these variables, the corresponding effect sizes were small.

Detailed descriptive statistics and results of the group comparisons are presented in Table 3.

### 3.3. Relationships Among Variables

Pearson correlation analyses revealed several significant associations among cancer-related self-efficacy, coping styles, and perceived stress (see Table 4). Cancer-related self-efficacy was positively associated with task-oriented coping, *r* = 0.343, 95% CI [0.167, 0.491], *p* < 0.001, and negatively associated with emotion-oriented coping, *r* = −0.443, 95% CI [−0.601, −0.263], *p* < 0.001. Furthermore, higher cancer-related self-efficacy was related to lower levels of perceived stress, *r* = −0.439, 95% CI [−0.597, −0.246], *p* < 0.001. No significant association was observed between cancer-related self-efficacy and avoidance-oriented coping, *r* = 0.108, 95% CI [−0.066, 0.283], *p* = 0.293.

Among coping styles, task-oriented coping was negatively associated with emotion-oriented coping, *r* = −0.441, 95% CI [−0.601, −0.244], *p* < 0.001, and with perceived stress, *r* = −0.324, 95% CI [−0.491, −0.124], *p* = 0.001. Emotion-oriented coping demonstrated the strongest association with perceived stress, *r* = 0.711, 95% CI [0.616, 0.793], *p* < 0.001, indicating that greater reliance on emotion-focused coping was related to substantially higher stress levels.

Avoidance-oriented coping was not significantly associated with cancer-related self-efficacy, task-oriented coping, emotion-oriented coping, or perceived stress (all *p*s > 0.05). Overall, these correlational findings are consistent with the hypothesized role of cancer-related self-efficacy in the associations between coping styles and perceived stress, which is examined formally in the mediation analyses reported in Section 3.4.

### 3.4. Mediation Analysis

Before conducting the mediation analyses, the assumptions of linearity, homoscedasticity, and the absence of influential observations were evaluated for each of the six regression equations comprising the three mediation models (i.e., the *a*-path and the combined *b*/*c′*-path for each coping style). Assumptions were assessed through visual inspection of residual and Q–Q plots, Cook’s distance, variance inflation factors (VIFs), and the Durbin–Watson statistic.

Visual inspection of the diagnostic plots indicated no substantial departures from linearity, homoscedasticity, or normality of residuals. Cook’s distance values were low across all models (maximum = 0.175; range = 0.10–0.18), indicating that no individual observation exerted an undue influence on the regression estimates. In the two-predictor regression models (coping style and cancer-related self-efficacy predicting perceived stress), VIF values ranged from 1.01 to 1.24 (tolerance = 0.80–0.99), well below conventional thresholds, indicating no evidence of multicollinearity.

The Durbin–Watson statistic ranged from 1.57 to 2.08 across models. A statistically significant result was observed only for the avoidance-oriented coping model (Durbin–Watson = 1.57, *p* = 0.028). However, because the Durbin–Watson test is intended to detect autocorrelation in temporally or spatially ordered observations, and the present study employed a cross-sectional design without a meaningful ordering of cases, this finding was not considered indicative of a substantive violation of regression assumptions. Detailed regression diagnostics for all six regression models are presented in Appendix B.

A series of simple mediation analyses (PROCESS Model 4; [41]) was conducted to examine whether cancer-related self-efficacy mediated the associations between coping styles and perceived stress. Task-oriented coping, emotion-oriented coping, and avoidance-oriented coping were examined as predictors, with cancer-related self-efficacy specified as the mediator and perceived stress as the outcome variable.

Task-oriented coping was positively associated with cancer-related self-efficacy, B = 0.25, SE = 0.07, *t* (95) = 3.56, *p* < 0.001, 95% CI [0.11, 0.39]. Cancer-related self-efficacy was negatively associated with perceived stress, B = −0.41, SE = 0.11, *t* (94) = −3.85, *p* < 0.001, 95% CI [−0.62, −0.20]. The total effect of task-oriented coping on perceived stress was significant, B = −0.26, SE = 0.08, *t* (95) = −3.33, *p* = 0.001, 95% CI [−0.42, −0.11]. After including cancer-related self-efficacy in the model, the direct effect of task-oriented coping on perceived stress remained significant, B = −0.16, SE = 0.08, *t* (94) = −2.03, *p* = 0.045, 95% CI [−0.31, −0.004]. The indirect effect of task-oriented coping on perceived stress through cancer-related self-efficacy was significant, B = −0.10, BootSE = 0.04, 95% CI [−0.19, −0.04]. These findings indicate that greater use of task-oriented coping was associated with higher cancer-related self-efficacy, which in turn was associated with lower perceived stress.

Emotion-oriented coping was negatively associated with cancer-related self-efficacy, B = −0.30, SE = 0.06, *t* (95) = −4.81, *p* < 0.001, 95% CI [−0.42, −0.18]. When emotion-oriented coping was included in the model, cancer-related self-efficacy was not significantly associated with perceived stress, B = −0.17, SE = 0.09, *t* (94) = −1.95, *p* = 0.054, 95% CI [−0.34, 0.003]. The total effect of emotion-oriented coping on perceived stress was positive and significant, B = 0.53, SE = 0.05, *t* (95) = 9.85, *p* < 0.001, 95% CI [0.42, 0.63]. After including cancer-related self-efficacy in the model, the direct effect of emotion-oriented coping on perceived stress remained significant, B = 0.48, SE = 0.06, *t* (94) = 8.09, *p* < 0.001, 95% CI [0.36, 0.59]. The indirect effect of emotion-oriented coping on perceived stress through cancer-related self-efficacy was not significant, B = 0.05, BootSE = 0.03, 95% CI [−0.01, 0.11].

Avoidance-oriented coping was not significantly associated with cancer-related self-efficacy, B = 0.11, SE = 0.10, *t* (95) = 1.06, *p* = 0.293, 95% CI [−0.10, 0.31]. Cancer-related self-efficacy was negatively associated with perceived stress when avoidance-oriented coping was included in the model, B = −0.48, SE = 0.10, *t* (94) = −4.69, *p* < 0.001, 95% CI [−0.68, −0.28]. The total effect of avoidance-oriented coping on perceived stress was not significant, B = −0.07, SE = 0.11, *t* (95) = −0.65, *p* = 0.515, 95% CI [−0.30, 0.15]. Furthermore, the indirect effect of avoidance-oriented coping on perceived stress through cancer-related self-efficacy was not significant, B = −0.05, BootSE = 0.04, 95% CI [−0.14, 0.03]. Thus, cancer-related self-efficacy did not mediate the relationship between avoidance-oriented coping and perceived stress (see Table 5).

Because no a priori power analysis was conducted before data collection, a post hoc sensitivity analysis was performed to characterize the minimum detectable effect size given the achieved sample size. For the two-predictor regression models (*n* = 97, α = 0.05, power = 0.80), the minimum detectable effect corresponded to Cohen’s *f*^2^ = 0.083 (approximately partial *r* = 0.28), representing a small-to-medium effect. Consequently, the study was adequately powered to detect effects of this magnitude or larger but may have lacked sufficient power to detect smaller effects. Therefore, the non-significant association observed for avoidance-oriented coping should be interpreted cautiously, as small effects cannot be ruled out.

## 4. Discussion

### 4.1. Coping Styles and Cancer-Related Self-Efficacy

The findings support H2a and H2b but not H2c. As predicted by H2a, task-oriented coping was positively associated with cancer-related self-efficacy. As predicted by H2b, emotion-oriented coping was negatively associated with cancer-related self-efficacy. Contrary to H2c, however, avoidance-oriented coping showed no significant association with cancer-related self-efficacy.

Drawing on Bandura’s [14] account of enactive mastery experience as a proposed source of self-efficacy beliefs, one plausible interpretation is that active, problem-focused coping tends to co-occur with greater confidence in managing cancer-related demands, whereas coping centered on emotional preoccupation tends to co-occur with less. This should be read as a theoretically motivated interpretation of the observed associations rather than as a demonstrated mechanism: the cross-sectional design cannot establish whether coping style precedes, follows, or is reciprocally related to self-efficacy beliefs over time. The direction and relative magnitude of these associations nonetheless align with prior cross-sectional findings in cancer patients and their spouses, where self-efficacy was moderately positively correlated with task-oriented coping and inversely correlated with emotion-oriented coping [29], and with the broader pattern linking adaptive, problem-oriented coping to higher self-efficacy across cancer populations [27].

The non-significant association between avoidance-oriented coping and cancer-related self-efficacy—contrary to H2c—is consistent with prior longitudinal evidence describing the link between self-efficacy and cognitive avoidance coping as notably weak [28]. It also fits the broader characterization of avoidance-oriented coping as a heterogeneous strategy, whose relationship with outcomes may depend on the controllability of the stressor and on the relative weight of its distraction- and disengagement-related components [25,26], rather than following a single, consistent association with personal resources such as self-efficacy. Taken together with the null association between avoidance-oriented coping and perceived stress reported below (Section 4.2), this suggests that avoidance-oriented coping occupied a comparatively marginal position within the broader network of associations among coping, self-efficacy, and stress in this sample, relative to task- and emotion-oriented coping.

### 4.2. Coping Styles, Cancer-Related Self-Efficacy, and Perceived Stress

Consistent with H1a and H1b, task-oriented coping showed a significant negative total association with perceived stress, while emotion-oriented coping showed a significant positive total association. These associations align in direction with those previously reported by our group in this same clinical sample in relation to pain [31] and disease appraisal [32], pointing to a consistent pattern linking coping style—particularly emotion-oriented coping—to indicators of psychological distress across several outcome variables measured in this population. Contrary to H1c, avoidance-oriented coping showed no significant total association with perceived stress, in line with the mixed pattern of associations reported elsewhere for avoidance-oriented coping in oncology samples [25,26].

The association between cancer-related self-efficacy and perceived stress (H3) was not uniform across the three parallel models. When considered alongside task-oriented or avoidance-oriented coping, cancer-related self-efficacy was significantly and negatively associated with perceived stress in both cases—a pattern consistent with self-efficacy’s documented association with lower distress in oncology populations more broadly [19,20,34]. When considered alongside emotion-oriented coping, however, this association was attenuated to a marginal, non-significant level. A plausible, purely descriptive explanation is that emotion-oriented coping and perceived stress share a substantial amount of negative-affect-related content, such that once emotion-oriented coping is entered into the model, little variance in perceived stress remains statistically associated with cancer-related self-efficacy. This should not be read as evidence that emotion-oriented coping “explains away” or causally supersedes self-efficacy; rather, it indicates that, within this cross-sectional dataset, the two constructs are correlated with overlapping portions of the variance in perceived stress, and their relative statistical contributions cannot be disentangled causally with the present design.

### 4.3. Cancer-Related Self-Efficacy as a Mediator (H4)

The clearest support for H4 was observed for the task-oriented pathway. Cancer-related self-efficacy was found to statistically mediate the association between task-oriented coping and perceived stress. Because the direct association between task-oriented coping and perceived stress remained statistically significant after accounting for self-efficacy, this pattern is consistent with partial rather than full statistical mediation: the total association between task-oriented coping and perceived stress appears to be distributed between a component shared with cancer-related self-efficacy and a component independent of it. Consistent with the framework outlined in the Introduction, this pattern is compatible with the proposal that active coping efforts and self-efficacy beliefs are correlated resources that are jointly associated with lower perceived stress, as would be expected under Conservation of Resources theory’s depiction of coping as a resource that co-occurs with, and may contribute to, the accumulation of further resources within a resource caravan [21]. This description remains a statistical, correlational characterization of the data; it does not establish that task-oriented coping generates self-efficacy, nor that self-efficacy in turn reduces stress, in a causal or temporal sense.

H4 was not supported for the emotion-oriented pathway. Although emotion-oriented coping was significantly associated with lower cancer-related self-efficacy, and although its total and direct associations with perceived stress were both substantial, the indirect association through cancer-related self-efficacy was not statistically significant. Together with the marginal association between self-efficacy and stress noted in this model (Section 4.2), this suggests that, in this sample, the statistical association between emotion-oriented coping and perceived stress runs largely outside of cancer-related self-efficacy—plausibly because emotion-oriented coping and perceived stress are themselves closely correlated constructs sharing overlapping affective content, leaving comparatively little residual, self-efficacy-associated variance to be statistically distinguished. H4 was similarly not supported for the avoidance-oriented pathway, consistent with the absence of a significant association between avoidance-oriented coping and either cancer-related self-efficacy or perceived stress in this sample.

Taken together, these results present a more differentiated pattern of association than the uniform mediating role initially proposed in H4: cancer-related self-efficacy appears as a statistical link between coping and perceived stress specifically in relation to task-oriented coping, whereas the association between emotion-oriented coping and perceived stress in this sample appears largely independent of self-efficacy, at least as captured by the present cross-sectional model.

### 4.4. Directionality and the Cross-Sectional Design

As outlined in the Introduction, the broader coping–self-efficacy literature is not unanimous regarding the direction of association: some studies report patterns consistent with self-efficacy as an antecedent of coping [27,28], others report patterns consistent with coping shaping self-efficacy [29], and at least one longitudinal study suggests the two may be mutually associated over time [28]. The present findings were obtained under a model in which coping styles were specified as the predictor and cancer-related self-efficacy as the statistical mediator; this specification reflects a theoretically motivated choice made prior to analysis rather than an empirically established causal ordering. Because the data are cross-sectional, they are equally compatible with self-efficacy preceding coping, with coping preceding self-efficacy, or with the two being reciprocally associated over the course of illness and treatment. The statistically significant indirect association observed for task-oriented coping should accordingly be interpreted as a cross-sectional, correlational pattern rather than as evidence of a directional or causal mechanism; no claim is made here that task-oriented coping produces, builds, or causes higher self-efficacy, or that self-efficacy in turn reduces perceived stress, only that these variables are statistically related to one another in the pattern described.

### 4.5. Interpreting Gender Differences in Study Variables

As outlined in the Introduction, potential gender differences in the study variables were examined separately through independent-samples *t*-tests rather than incorporating gender as a covariate or moderator, given the marginal, non-significant moderating role of gender identified in our prior work on this same sample [31].

Consistent with the broader literature associating female gender in cancer populations with higher emotional distress and greater reliance on emotion-focused responses to illness [8,9,10], women in the present sample reported higher levels of perceived stress and greater use of emotion-oriented coping than men. Conversely, no significant gender differences emerged for cancer-related self-efficacy, task-oriented coping, or avoidance-oriented coping.

From a descriptive perspective, it is notable that the two variables differing significantly by gender—perceived stress and emotion-oriented coping—correspond to the statistical pathway that operated largely independently of cancer-related self-efficacy in our mediation analysis. In contrast, cancer-related self-efficacy and task-oriented coping—the variables constituting the only significant indirect pathway in the model—showed no significant differences between men and women. This co-occurrence is offered strictly as an empirical observation.

## 5. Limitations and Future Research

Several methodological limitations should be considered when interpreting the present findings. Most importantly, the cross-sectional design does not allow for temporal ordering or causal inference among coping styles, cancer-related self-efficacy, and perceived stress. The statistical mediation reported here reflects a pattern of association, not a demonstrated causal pathway; the model direction—coping styles as predictors and self-efficacy as mediator—was chosen on theoretical grounds prior to analysis, but the data are equally compatible with the reverse or a reciprocal relationship unfolding over time [28]. Prospective, longitudinal designs are needed to adjudicate between these possibilities.

The sample was relatively modest and drawn from a single clinical setting, limiting generalizability to broader or more heterogeneous lung cancer populations. The present analysis also draws on the same clinical sample examined in three previous reports from our group, focused on social support and disease appraisal [33], cognitive appraisal and coping styles [32], and coping styles and pain [31]; the current study extends this work by introducing cancer-related self-efficacy as a previously untested variable. Because these studies share a common sample, consistent findings across them—particularly the role of emotion-oriented coping—should be regarded as internally consistent rather than as independent replications, and confirmation in an independent sample is needed. Although the post hoc sensitivity analysis indicated adequate power to detect small-to-medium effects, the study may have been underpowered to detect small associations. Consequently, the non-significant findings for avoidance-oriented coping should be interpreted with caution.

Data collection relied entirely on self-report questionnaires, which may have introduced common-method variance. Clinically relevant characteristics—including disease stage, time since diagnosis, treatment phase, and comorbidity burden—were not incorporated into the model and represent a potential source of confounding that the present design cannot rule out.

Future research would benefit from prospective designs tracking how coping styles, cancer-related self-efficacy, and perceived stress covary across the course of diagnosis and treatment, and from models incorporating depressive and anxiety symptoms, objective social support, and clinical disease characteristics. Given the substantial physical burden of lung cancer, future studies should also consider assessing pain, which may share variance with perceived stress in this population [7]. Socioeconomic and environmental factors such as place of residence—previously found to moderate coping–outcome associations in this same sample [31]—may further clarify how contextual factors relate to the personal resources examined here.

## 6. Conclusions

The present study demonstrates that coping styles are associated with perceived stress among patients with lung cancer, and that this association differs by coping style: task-oriented coping was linked to lower perceived stress, and emotion-oriented coping to higher perceived stress, while avoidance-oriented coping showed no association with stress in this sample. The study further shows that cancer-related self-efficacy accounts, in part, for the association between task-oriented coping and perceived stress, indicating that self-efficacy is one statistical pathway—though not the only one—through which active coping relates to lower stress. By contrast, the association between emotion-oriented coping and perceived stress operates largely independently of self-efficacy, suggesting that these two coping styles are linked to perceived stress through at least partly distinct mechanisms.

These findings extend prior work from our own group on this same clinical sample, which established coping style—and emotion-oriented coping in particular—as a consistent correlate of psychological distress in lung cancer patients [31,32], by identifying cancer-related self-efficacy as a previously untested variable that helps account for part of this pattern. As the design is cross-sectional, the study demonstrates a pattern of statistical association rather than a causal or temporally ordered mechanism, and does not establish that changing coping style or self-efficacy would alter perceived stress; this remains a question for future intervention research.

## Figures and Tables

**Table 1:** Characteristics of the Study Sample (*n* = 97).

Variable		*n*	%
Sex	Male	50	51.5
	Female	47	48.5
	Total	97	100.0
Smoking tobacco	Yes	48	49.5
	No	49	50.5
	Total	97	100.0
Type of cancer	Non-small-cell cancer	81	83.5
	Small-cell cancer	16	16.5
	Total	97	100.0
Marital status	Married	68	70.1
	Widowed	19	19.6
	Divorced	6	6.2
	Single	4	4.1
	Total	97	100.0
Place of residence	Village	38	39.2
	Small town	18	18.6
	Medium-sized city	14	14.4
	Big city	26	26.8
	Total	96	99.0
	Missing	1	1.0
Professional status	Retirement pension	61	62.9
	Professionally active	18	18.6
	Disability pension	11	11.3
	Sick leave	5	5.2
	Total	95	97.9
	Missing	2	2.1
Treatment used ^a^	Chemotherapy	93	95.9
	Radiotherapy	16	16.5
	Surgical treatment	9	9.3
Number of chemotherapy cycles	1–3	36	39.6
	4–7	46	47.4
	8 or more	9	9.3
	Total	91	93.8
	Missing	6	6.2
Time since diagnosis	0–54 months	23	27.8
	55–68 months	52	53.6
	69 months or more	9	9.3
	Total	84	90.7
	Missing	13	13.4

^a^ Categories are not mutually exclusive, as patients could receive more than one treatment modality; percentages do not sum to 100%.

**Table 2:** Descriptive Statistics for All Study Variables (*n* = 97).

Variable	*M*	*SD*	*Mdn*	*Min*	*Max*	*Sk*	*Kurt*
Cancer-related self-efficacy	29.24	6.03	30.00	10.00	40.00	−0.76	1.87
Task-oriented coping	57.30	8.18	57.00	36.00	79.00	0.38	0.42
Emotion-oriented coping	38.66	8.91	39.00	23.00	63.00	0.27	−0.20
Avoidance-oriented coping	44.89	6.00	45.00	31.00	64.00	0.03	0.25
Perceived stress	17.33	6.60	17.00	2.00	37.00	0.16	0.17

Note. *M* = mean; *SD* = standard deviation; *Mdn* = median; *Sk* = skewness; *Kurt* = kurtosis. Skewness and kurtosis values indicated no substantial deviations from normality across the study variables.

**Table 3:** Gender Differences in Study Variables.

Variable	Women (*n* = 47) *M (SD)*	Men (*n* = 50) *M (SD)*	*t* (df)	*p*	Cohen’s *d*
Cancer-related self-efficacy	28.24 (6.06)	30.18 (5.92)	−1.59 (95)	0.115	−0.32
Perceived stress	19.57 (6.88)	15.22 (5.63)	3.42 (95)	<0.001	0.70
Task-oriented coping	56.35 (8.26)	58.20 (8.08)	−1.11 (95)	0.268	−0.23
Emotion-oriented coping	41.80 (8.96)	35.71 (7.86)	3.56 (95)	<0.001	0.72
Avoidance-oriented coping	44.11 (5.31)	45.62 (6.55)	−1.25 (95)	0.216	−0.25

Note. *M* = mean; *SD* = standard deviation. Positive values of Cohen’s *d* indicate higher scores among women, whereas negative values indicate higher scores among men. Statistically significant differences (*p* < 0.05) were observed for perceived stress and emotion-oriented coping.

**Table 4:** Pearson Correlations and 95% Confidence Intervals for Study Variables (*n* = 97).

Variable	1	2	3	4
1. Cancer-related self-efficacy	-			
2. Task-oriented coping	0.343 *** [0.167, 0.491]	-		
3. Emotion-oriented coping	−0.443 *** [−0.601, −0.263]	−0.441 *** [−0.601, −0.244]	-	
4. Avoidance-oriented coping	0.108 [−0.066, 0.283]	0.049 [−0.173, 0.276]	0.017 [−0.192, 0.216]	-
5. Perceived stress	−0.439 *** [−0.597, −0.246]	−0.324 ** [−0.491, −0.124]	0.711 *** [0.616, 0.793]	−0.067 [−0.287; 0.151]

Note. Values are Pearson correlation coefficients (r). Values in brackets represent 95% bootstrap confidence intervals based on 1000 resamples. ** *p* < 0.01, *** *p* < 0.001.

**Table 5:** Mediation Analyses Examining Cancer-Related Self-Efficacy as a Mediator of the Associations Between Coping Styles and Perceived Stress.

Path/Effect	B	SE	t	*p*	95% CI
**Task-oriented coping → Cancer-related self-efficacy → Perceived stress**					
Task-oriented coping → CRSE (*a*)	0.25	0.07	3.56	<0.001	[0.11, 0.39]
CRSE → Perceived stress (*b*)	−0.41	0.11	−3.85	<0.001	[−0.62, −0.20]
Total effect (*c*)	−0.26	0.08	−3.33	0.001	[−0.42, −0.11]
Direct effect (*c*′)	−0.16	0.08	−2.03	0.045	[−0.31, −0.004]
Indirect effect via CRSE	−0.10	0.04	-	-	[−0.19, −0.04]
**Emotion-oriented coping → Cancer-related self-efficacy → Perceived stress**					
Emotion-oriented coping → CRSE (*a*)	−0.30	0.06	−4.81	<0.001	[−0.42, −0.18]
CRSE → Perceived stress (*b*)	−0.17	0.09	−1.95	0.054	[−0.34, 0.003]
Total effect (*c*)	0.53	0.05	9.85	<0.001	[0.42, 0.63]
Direct effect (*c*′)	0.48	0.06	8.09	<0.001	[0.36, 0.59]
Indirect effect via CRSE	0.05	0.03	-	-	[−0.01, 0.11]
**Avoidance-oriented coping → Cancer-related self-efficacy → Perceived stress**					
Avoidance-oriented coping → CRSE (*a*)	0.11	0.10	1.06	0.293	[−0.10, 0.31]
CRSE → Perceived stress (*b*)	−0.48	0.10	−4.69	<0.001	[−0.68, −0.28]
Total effect (*c*)	−0.07	0.11	−0.65	0.515	[−0.30, 0.15]
Direct effect (*c*′)	−0.02	0.10	−0.21	0.832	[−0.23, 0.18]
Indirect effect via CRSE	−0.05	0.04	-	-	[−0.14, 0.03]

Note. CRSE = cancer-related self-efficacy; PSS = Perceived Stress Scale. Indirect effects were estimated using 5000 bootstrap samples. An indirect effect was considered statistically significant when the 95% bootstrap confidence interval did not include zero.

**Figure A1:**
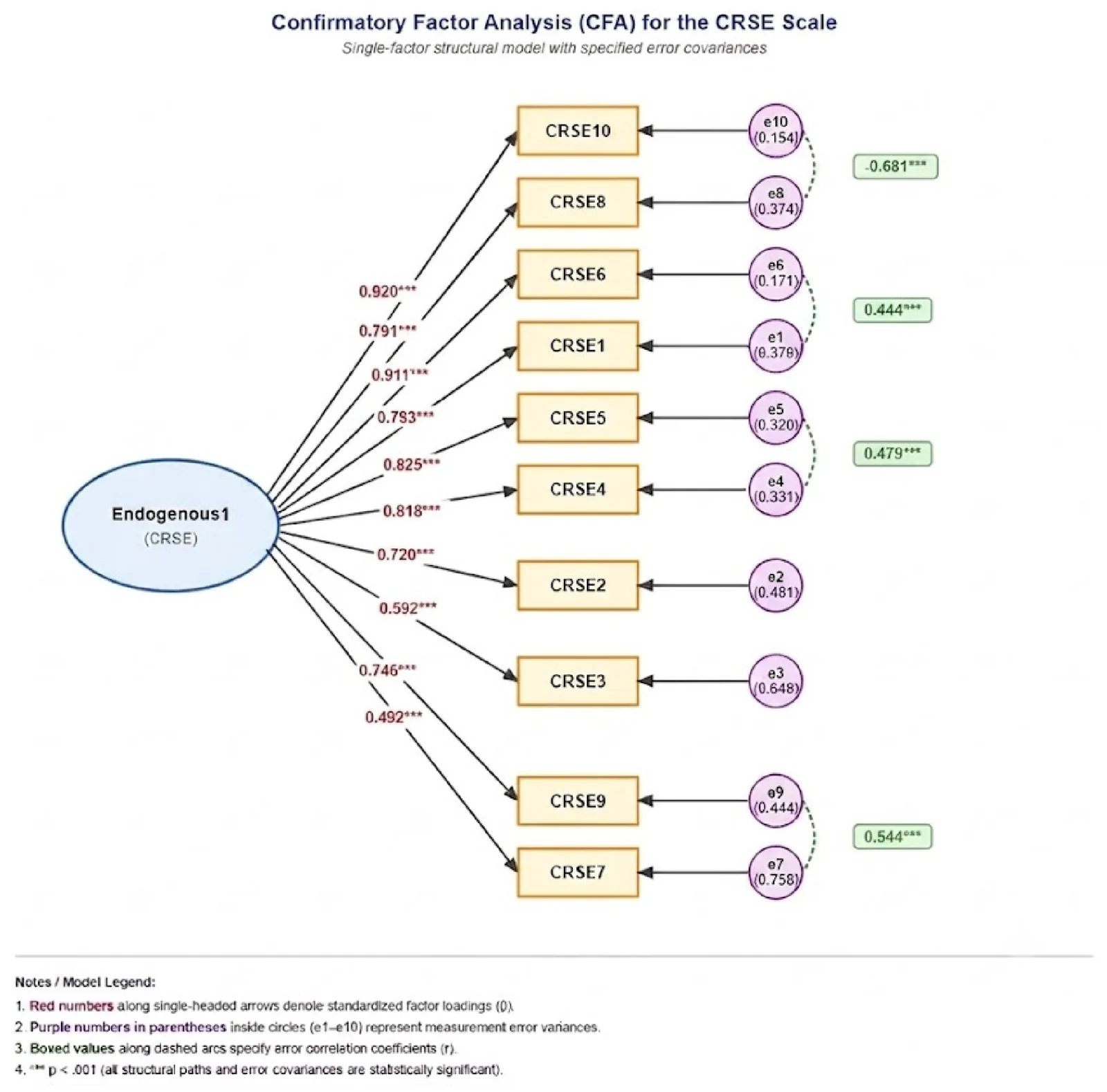
Confirmatory factor analysis (single-factor model) of the CRSE scale, showing standardized factor loadings (red) and error variances (purple) for items CRSE1–CRSE10.

## Data Availability

The data presented in this study are available on request from the corresponding author. The data are not publicly available due to privacy and ethical restrictions, as the datasets contain sensitive clinical and psychological information regarding patients undergoing oncological treatment.

## Abbreviations

The following abbreviations are used in this manuscript:

GSES	Generalized Self-Efficacy Scale
CRSE	Cancer-Related Self-Efficacy
CISS	Coping Inventory for Stressful Situations
TOS	task-oriented style
EOS	emotion-oriented style
AOS	avoidance-oriented style
PSS	Perceived Stress Scale
LC	Lung Cancer
M	Mean
SD	Standard deviation
SE	Standard Error
Mdn	Median
Sk	Skewness
Kurt	Kurtosis
CI	Confidence interval

## Appendix A. Wording of the Modified Cancer-Related Self-Efficacy Scale Items

**Table A1:** Polish and English wording of the 10 items adapted from the Generalized Self-Efficacy Scale [35,36] for the present study, modified to refer specifically to coping with cancer-related situations.

Item	Polish (Administered Version)	English
CRSE1	Zazwyczaj jestem w stanie rozwiązać trudne problemy związane z chorobą nowotworową, jeśli tylko wystarczająco się postaram.	I can always manage to solve difficult problems related to my cancer if I try hard enough.
CRSE2	Nawet gdy ktoś mi się sprzeciwia lub napotykam trudności związane z chorobą nowotworową, jestem w stanie znaleźć sposób na osiągnięcie tego, czego chcę.	If someone opposes me, I can find the means and ways to get what I want despite my cancer.
CRSE3	Z łatwością potrafię trzymać się swoich celów i je osiągać, mimo wyzwań związanych z chorobą nowotworową.	It is easy for me to stick to my aims and accomplish my goals despite my cancer.
CRSE4	Jestem przekonany(-a), że skutecznie poradził(a)bym sobie z nieoczekiwanymi sytuacjami związanymi z chorobą nowotworową.	I am confident that I could deal efficiently with unexpected events related to my cancer.
CRSE5	Dzięki swojej pomysłowości i zaradności wiem, jak poradzić sobie z nieprzewidzianymi sytuacjami związanymi z chorobą nowotworową.	Thanks to my resourcefulness, I know how to handle unforeseen situations related to my cancer.
CRSE6	Jestem w stanie rozwiązać większość problemów związanych z chorobą nowotworową, jeśli tylko włożę w to odpowiednio dużo wysiłku.	I can solve most problems related to my cancer if I invest the necessary effort.
CRSE7	Kiedy zmagam się z przeciwnościami związanymi z chorobą nowotworową, jestem w stanie zachować spokój, gdyż mogę polegać na swoich umiejętnościach radzenia sobie.	I can remain calm when facing difficulties related to my cancer because I can rely on my coping abilities.
CRSE8	Kiedy zmagam się z problemem związanym z chorobą nowotworową, zazwyczaj jestem w stanie znaleźć kilka sposobów jego rozwiązania.	When I am confronted with a problem related to my cancer, I can usually find several solutions.
CRSE9	Gdy mam kłopoty związane z chorobą nowotworową, to zazwyczaj jestem w stanie wymyślić sposób, jak z nich wyjść.	If I am in trouble because of my cancer, I can usually think of a solution.
CRSE10	Zazwyczaj jestem w stanie poradzić sobie z tym, co niesie ze sobą choroba nowotworowa.	I can usually handle whatever comes my way because of my cancer.

Note. Item order and standardized factor loadings from the confirmatory factor analysis (see Table A2, Section 2.2) correspond to this numbering (CRSE1–CRSE10).

**Table A2:** Confirmatory Factor Analysis Loadings for the Modified CRSE Scale.

Item	Standardized Loading (β)	SE	z	*p*
CRSE10	0.920	—	—	—
CRSE6	0.911	0.068	14.85	<0.001
CRSE9	0.746	0.075	9.63	<0.001
CRSE1	0.793	0.093	10.77	<0.001
CRSE8	0.791	0.089	8.49	<0.001
CRSE5	0.825	0.081	11.70	<0.001
CRSE4	0.818	0.082	11.50	<0.001
CRSE2	0.720	0.086	9.08	<0.001
CRSE3	0.592	0.097	6.75	<0.001
CRSE7	0.492	0.085	5.30	<0.001

Note. Maximum likelihood estimation; *n* = 97. CRSE10 was fixed as the marker item (unstandardized loading = 1.00). Model included four correlated residual pairs: CRSE4↔CRSE5, CRSE7↔CRSE9, CRSE1↔CRSE6, CRSE8↔CRSE10.

## Appendix B

**Table A3:** Regression diagnostics for the mediation models.

Outcome	Predictor(s)	R^2^	F (df1, df2)	Cook’s Distance (max)	VIF	Tolerance	Durbin–Watson	*p* (D–W)
CRSE	Task-oriented style	0.117	12.60 (1, 95) ***	0.121	1.00	1.00	1.81	0.350
PSS	TOS + CRSE	0.227	13.80 (2, 94) ***	0.175	1.13	0.883	1.67	0.122
CRSE	Emotion-oriented style (EOS)	0.196	23.20 (1, 95) ***	0.147	1.00	1.00	1.83	0.444
PSS	EOS + CRSE	0.524	51.80 (2, 94) ***	0.097	1.24	0.804	2.08	0.688
CRSE	Avoidance-oriented style (AOS)	0.012	1.12 (1, 95)	0.101	1.00	1.00	1.92	0.700
PSS	AOS + CRSE	0.193	11.20 (2, 94) ***	0.120	1.01	0.988	1.57	0.028

Note. *n* = 97. CRSE = cancer-related self-efficacy; PSS = perceived stress. TOS = task-oriented style; EOS = emotion-oriented style; AOS = avoidance-oriented style. VIF values below 5 and Cook’s distance values below 1.0 indicate no evidence of problematic multicollinearity or influential observations. *** *p* < 0.001.

**Table A4:** STROBE.

Section/Topic	Item	STROBE Item Description	Reported on Page/Section
Title and abstract			
	1a	Indicate the study’s design with a commonly used term in the title or the abstract.	Title (“Cross-Sectional Mediation Analysis”); Abstract (Methods)
	1b	Provide in the abstract an informative and balanced summary of what was done and what was found.	Abstract (Objective, Methods, Results, Conclusions)
Introduction			
Background/rationale	2	Explain the scientific background and rationale for the investigation being reported.	Section 1 (Introduction)
Objectives	3	State specific objectives, including any prespecified hypotheses.	Section The Present Study (The Present Study; Hypotheses H1–H4)
Methods			
Study design	4	Present key elements of study design early in the paper.	Abstract (Methods); Section 2.1 (Participants and Procedure)
Setting	5	Describe the setting, locations, and relevant dates, including periods of recruitment and data collection.	Section 2.1 (Lublin, Poland; October 2016–September 2017)
Participants	6a	Give the eligibility criteria and the sources and methods of selection of participants.	Section 2.1 (Clinical sample, diagnosed with lung cancer; no strict exclusion criteria applied)
Variables	7	Clearly define all outcomes, exposures, predictors, potential confounders, and effect modifiers.	Section 2.1 and Section 2.3; Table 1
Data sources/ measurement	8	Give sources of data and details of methods of assessment for each variable.	Section 2.3 (Measures: CISS, adapted GSES/CRSE, PSS-10); Section 2.1
Bias	9	Describe any efforts to address potential sources of bias.	Section 2.1 (Face-to-face assistance to reduce completion bias); Section 5 (Limitations)
Study size	10	Explain how the study size was arrived at.	Section 2.1 (97/100 participants); Section 2.3 and Section 5 (Post hoc sensitivity power analysis, *n* = 97)
Quantitative variables	11	Explain how quantitative variables were handled in the analyses.	Section 2.3 (Continuous scale scores used; no categorization applied)
Statistical methods	12a	Describe all statistical methods, including those used to control for confounding.	Section 2.3 (Pearson correlations, independent *t*-tests, PROCESS Model 4 mediation)
	12b	Describe methods used to examine subgroups and interactions.	Section 2.3; Section 3.2 (Gender comparisons using independent-samples *t*-tests)
	12c	Explain how missing data were addressed.	Section 2.2 (Missing Data—pairwise deletion used due to ~2% minimal missingness)
	12d	If applicable, describe analytical methods taking account of sampling strategy.	Not applicable (Consecutive clinical sample)
	12e	Describe any sensitivity analyses.	Section 2.3 and Section 5 (Post hoc sensitivity power analysis for minimum detectable effect size)
Results			
Participants	13a	Report numbers of individuals at each stage of the study.	Section 2.1 (100 invited, 3 declined, 97 analyzed)
	13b	Give reasons for non-participation at each stage.	Section 2.1 (3 patients declined participation)
	13c	Consider use of a flow diagram.	Not included (Participant flow described in text, Section 2.1)
Descriptive data	14a	Give characteristics of study participants and information on exposures and potential confounders.	Table 1 (Sociodemographic & clinical data); Table 2 (Descriptive statistics)
	14b	Indicate number of participants with missing data for each variable.	Table 1; Section 2.2
Outcome data	15	Report outcome events or summary measures.	Table 2 (Means, SDs, Ranges) & Table 3 (Gender comparisons)
Main results	16a	Give unadjusted estimates and, if applicable, adjusted estimates with precision (e.g., 95% CI).	Table 4 (Pearson r with 95% bootstrap CI); Table 5 (Mediation regression models with 95% bootstrap CIs)
	16b	Report category boundaries when continuous variables were categorized.	Not applicable
	16c	If relevant, translate estimates into absolute risk.	Not applicable
Other analyses	17	Report other analyses done—e.g., analyses of subgroups, interactions, and sensitivity analyses.	Section 3.2 (Gender differences); Section 3.4 and Appendix B (Regression diagnostics & sensitivity power analysis)
Discussion			
Key results	18	Summarise key results with reference to study objectives.	Section 4.1, Section 4.2 and Section 4.3; Section 6 (Conclusions)
Limitations	19	Discuss limitations of the study, including potential bias or imprecision.	Section 5 (Cross-sectional design, single-center, lack of clinical stage covariates, self-report)
Interpretation	20	Give an overall cautious interpretation of results.	Section 4.4 and Section 5, and 6
Generalisability	21	Discuss the generalisability (external validity) of the study results.	Section 5 (Limitations regarding sample size and single-center clinical recruitment)
Other information			
Funding	22	Give the source of funding and the role of the funders.	Funding Statement (“This research received no external funding”)
